# Beyond Gene Delivery: Strategies to Engineer the Surfaces of Viral Vectors

**DOI:** 10.3390/biomedicines1010003

**Published:** 2013-12-04

**Authors:** Cristian Capasso, Mari Hirvinen, Vincenzo Cerullo

**Affiliations:** Laboratory of Immunovirotherapy, Division of Biopharmaceutics and Pharmacokinetics, Faculty of Pharmacy, University of Helsinki, Helsinki 00760, Finland; E-Mails: cristian.capasso@helsinki.fi (C.C.); mari.hirvinen@helsinki.fi (M.H.)

**Keywords:** viral vectors, gene therapy, surface modification, polymers, peptides

## Abstract

Viral vectors have been extensively studied due to their great transduction efficiency compared to non-viral vectors. These vectors have been used extensively in gene therapy, enabling the comprehension of, not only the advantages of these vectors, but also the limitations, such as the activation of the immune system after vector administration. Moreover, the need to control the target of the vector has led to the development of chemical and non-chemical modifications of the vector surface, allowing researchers to modify the tropism and biodistribution profile of the vector, leading to the production of viral vectors able to target different tissues and organs. This review describes recent non-genetic modifications of the surfaces of viral vectors to decrease immune system activation and to control tissue targeting. The developments described herein provide opportunities for applications of gene therapy to treat acquired disorders and genetic diseases and to become useful tools in regenerative medicine.

## 1. Introduction

Knowledge of viral structures and infection mechanisms has paved the way for a deeper understanding of virus interactions with the human body. This information has become extremely important as viruses are being used as new vectors, capable of delivering transgenes, *in vitro* and *in vivo*, to tissues and cells. Cloning techniques have enabled the modification of different wild type viruses, such as adenoviruses, lentiviruses and retroviruses, herpes simplex viruses, and many others, to produce viral vectors for gene therapy [1,2,3]. First, viruses were genetically modified; in particular, genes involved in the replication cycle were deleted and/or replaced. These modifications had two consequences: (i) larger expression cassettes were inserted into viral genomes, and (ii) replication-defective vectors (RDV) and/or conditionally replicating vectors were produced.

The production of vectors unable to replicate proved to be extremely important in lowering vector immunogenicity. A clear example of this reduced immunogenicity is third generation adenoviral vectors, also called helper-dependent adenoviral vectors [4]. Most of the genome of these vectors is deleted and the resulting vectors are called “gutless-vectors”; hence, this type of adenoviral vector is not able to replicate and avoids the chronic toxicity that is typically related to protein expression of viral genes [5]. Lentiviral vectors have also been engineered to make these vectors unable to replicate once integrated into the host genome [6,7].

Modifying the viral genome by deleting genes essential for the replication process represented a quantum leap in gene therapy and provided researchers safer and more reliable vectors. Nevertheless, these new vectors exhibited limitations when applied to *in vivo* experimentation protocols. Even without viral gene expression, some immunological response was elicited anyway [8]. In fact, the interactions between the injected viral vector and molecular receptors responsible for the innate immune response [9], such as Toll-like receptors [10,11,12,13,14], are the basis for the acute toxicity observed *in vivo*, which is not related to viral replication but to the mere presence of the vector in the organism [8,15]. Therefore, viral vector surfaces were engineered for decreased visibility to the immune system. Viruses were masked to reduce interactions with pattern recognition receptors (PPRs) that are responsible for the innate immune response [16,17], and this strategy has successfully extended transgene expression and lowered anti-vector immune response.

Moreover, modifications of the virus structure and surface enable the control of viral biodistribution. To date, adenoviruses have been the most investigated type of virus due to the natural liver tropism of these viruses. Nevertheless, even if liver targeting was desirable, the need to target other organs has led to efforts to de-target adenoviral vectors. Liver de-targeting, with consequent controlled re-targeting of other tissues has been achieved through viral protein modification [18,19] or the conjugation of vectors with peptides and molecular adaptors.

## 2. Fighting the Immune Response

### 2.1. Polyethylene Glycol and a New Generation of Polymers

**Figure 1:**
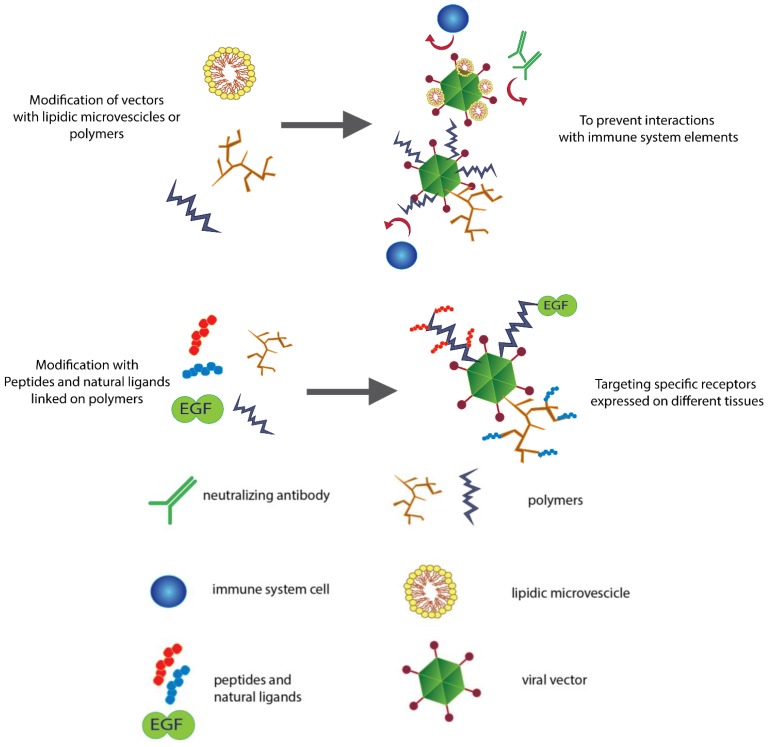
Modifying the surfaces of viral vectors for different purposes. Shielding viral vectors with polymers and lipidic vesicles typically results in the reduced immunogenicity and increased persistence of the vectors in the blood stream. Biomaterials and polymers represent a physical barrier that not only prevents neutralization from antibodies but also impairs the uptake of the vector by macrophages and antigen presenting cells (APC). Polymers can also be linked with peptides and ligands that target a specific receptor, changing the vector tropism and allowing efficient re-targeting.

The immune response elicited by viral vectors has limited the use of gene therapy in clinical trials [8,20,21]. Pre-existing immunity because of neutralizing antibodies and the interactions of viral capsids with PRRs activates the innate immune response [22], limiting the success rate of therapeutic approaches. To address this issue, viral vectors have started to be modified to reduce the physical interaction of these vectors with immune system elements (Figure 1). 

Polyethylene glycol (PEG), a polymer able to increase the half-life of many different drugs and reduce their immunogenicity [23], is commonly used to modify the viral surface. PEGylation is able to increase transduction efficiency of viral vectors in mice [24] and to prolong the circulation time of these vectors [25,26]. More specifically, in murine models, the half-life of systemically injected unmodified adenovirus is less than 2 min [27], while the attachment of PEG molecules to these viruses reduces viral clearance by a factor of four. Furthermore, adenovirus PEGylation is able to reduce the immune response against the vector [28,29]. The production of inflammatory cytokines (IL-6, IL-12, and TNFα) in response to vector administration is greatly affected by vector PEGylation, which is actually able to drastically lower the levels of pro-inflammatory cytokines in blood [24]. Moreover, the intravenous injection of PEGylated Ad vectors also resulted in significantly fewer Ad-specific cytotoxic T lymphocytes in comparison to unmodified Ad- and TH1-mediated responses, which also exhibited a marked decrease upon delivery of PEGylated Ad vectors. Interestingly, TH2-mediated responses remained unchanged or increased slightly [30]. In addition, PEGylation reduced the production of anti-Adenovirus antibodies (anti-Adv Abs) *in vivo*: Anti-Adv Ig-G and anti-Adv Ig-M serum levels of animals treated with PEGylated Ad vectors were greatly reduced in comparison to animals treated with naked adenovirus [31]. The PEG coating also helps overcome pre-existing immunity, shielding viral vectors from neutralizing antibodies and preserving transduction *in vitro* [32] and *in vivo* [24]. PEGylation of lentiviral vectors has proved to successfully protect these vectors from inactivation by human serum complement [33].

To improve the coating system, other polymers have been developed to engineer the surface of the viral vectors. Cationic polymers have also been used in combination with PEG, such as PEG-poly-L-lysine, which is a multi-block copolymer based on poly(L-lysine) and PEG. This interesting compound not only features low toxicity *in vitro* but this material is also biodegradable under physiological conditions: degradation of the copolymer has been reported within 72 h [34]. APC, a cationic PEG derivative, protects adenoviruses from neutralizing antibodies (NAbs). In fact, exposure to NAbs does not ablate the transduction activity of the APC-coated adenovirus [35] *in vitro*. The conjugation of *N*-[2-hydroxypropyl] methacrylamide (HPMA) copolymers to the surfaces of vectors represents another example of how a polymer coating can mask viral vectors from antibodies. An HPMA coating results in an increased half life, lower NAbs binding and decreased interaction with complement receptor 1 (CR1) on human erythrocytes [36], which are well known factors for the persistence in blood of viral vectors as well as inefficient tissue penetration. Therefore, the protection of vectors from NAbs due to an HPMA copolymer coating results in efficient transduction of these vectors to Coxsackievirus and adenovirus receptor (CAR)-positive and CAR-negative cells, even in the presence of NAbs [37]. Arginine-grafted bioreducible polymers (ABPs) can also be conjugated to the surfaces of viral vectors and decrease the production of pro-inflammatory cytokines. ABP conjugation to an oncolytic adenovirus significantly lowers IL-6 production *in vitro*, using RAW 264.7 macrophages, and *in vivo* [38].

### 2.2. Lipids Can Hide Vectors

In addition to polymers, lipids have also been used to improve the safety of viral vectors. Cationic liposomes are artificially generated lipid vesicles frequently used for DNA or drug delivery and have been widely used in fundamental molecular biology and for gene therapy as a non-viral vector. Small unilamellar vesicles (SUV) can be complexed to adenoviruses with a simple mixing reaction (30 min at 37 °C) [39]. In mice injected with Adenovirus/SUV complexes, the levels of anti-adenoviral vector antibodies are 6.5-fold lower than those from mice injected with the naked vector; thus, these polymer-coated vectors are less immunogenic. Moreover, SUV-conjugated Ad vectors are less susceptible to inactivation by neutralizing antibodies, allowing the successful re-administration of the adenoviral vector to mice [39].

Lipidic envelopes can also be used as shields for viral vectors to lower the immune response. DOTAP:CHOL and DOPE:CHEMS are, respectively, cationic and anionic lipid envelopes able to decrease the production of NAbs after intra-venous injection [40].

In conclusion, following the example of PEG, new generations of compounds and polymers have been developed to bind to capsid proteins and act as physical barriers to interactions between the immune system elements and viral proteins, resulting in less immunogenic vectors for gene therapy.

## 3. Controlling the Target

More than a decade ago, CAR was identified as the primary cell surface component to interact with these adenoviruses and the key for the entry of adenovirus 2 and 5 serotypes into permissive cells [41]. Along with alpha V integrin, these molecules were believed to be responsible for the observed liver tropism of adenoviral vectors *in vivo*. Furthermore, other viruses have been studied to understand the mechanisms behind their tropisms: vesicular stomatitis virus (VSV) shows neuronal tropisms due to glycoprotein (G) and matrix protein (M) [42]; nevertheless, this virus is also able to transduce into a great variety of tissues by binding the LDL receptor [43]. Similarly, human immunodeficiency virus (HIV) uses chemokine receptor CCR5 on lymphocytes for its entry into cells [44]. Even if the presence of a receptor on target cells was the only factor required for the entry of viruses, there has been an interesting exception to this rule: adenoviruses.

Once the elements on the adenovirus surface responsible for cell entry (fiber and an RGD motif in the penton base) were identified, several research groups began to modify capsid proteins to avoid liver transduction and to achieve the successful re-targeting of adenoviral vectors. Interestingly, adenoviruses with mutated fibers and an RGD motif deletion continued to show high liver transduction and liver accumulation [45]. These findings suggested that other factors were most likely involved in the tropism of adenoviral vectors, and later, coagulation factor X (FX) was identified as the key element for liver targeting of adenoviral vectors [46]. The idea that other elements, which do not belong to the structure of the virus, could affect viral tropism has been very attractive to researchers and a great number of strategies have been developed to modify the surface of viral vectors for re-targeting.

In this review, we have already discussed PEGylation and its importance in preventing capsid-mediated toxicity. Moreover, we would like to highlight that PEGylation may also influence cell entry and tissue targeting. In fact, depending on the size of the PEG molecules, the surface of the viruses can change drastically and alterations in the transduction of cells *in vitro* and *in vivo* are commonly observed. In addition, anatomical and physiological species differences should be taken into account when using PEGylated vectors: in rodents PEGylated vectors show similar transduction in comparison to unmodified viruses, however, in non-human primates, PEGylation causes a decrease in transduction [47].

The decreased transduction observed in different *in vitro* and *in vivo* experiments is not only related to the increased size of the modified vectors but also to the non-specific nature of the PEGylation process because controlling the site of attachment is not possible. PEG molecules can cover areas of the capsid that are necessary for cell entry. Hence, novel strategies have been developed to control the attachment of PEG molecules on the surface of adenoviruses. An interesting example is represented by the use of the FX as a PEG “guide”. In fact, due to the specific interaction between FX and hexon protein, FX can be used as an adapter to avoid uncontrolled coating. Specifically, FX is first PEGylated and then mixed with naked adenovirus [48].

### 3.1. Passive Re-Targeting with Lipids

High uptake of adenoviral vectors by the liver could be observed as an obstacle to the development of therapies. Additionally, avoiding excessive retention by the liver is an important reason to de-target viral vectors. The construction of lipidic envelopes constitutes a strategy to engineer viral surfaces to control the biological properties of these surfaces. In fact, differences in the surface potential could lead to drastic changes in transduction. Positive or negative surface envelopes seem to block the interaction with the CAR receptor. Nevertheless, cationic envelopes are still able to enhance vector uptake by binding the enveloped virus to the negatively charged cell membrane, but the presence of the envelope disrupts normal endosomal trafficking and leads to no transgene expression *in vitro* [39]. However, cationic non-pH sensitive envelopes (DOTAP:CHOL) and anionic pH-sensitive envelopes (DOPE:CHEMS) can be used *in vivo* to avoid liver uptake because, when injected intravenous (i.v.), these enveloped vectors show reduced liver accumulation [40]. Nano-engineering artificial envelopes around adenoviral vectors have also been reported to result in an enhanced ability to penetrate into tumor spheroid cell culture [39].

### 3.2. Passive Re-Targeting with Polymers and Magnetic Particles

The polymer coating of viral particles can also lead to liver de-targeting. In fact, PEGylation with either low or high molecular weight PEG has been reported to reduce transduction of cells *in vitro* [49,50,51]. Interestingly, even if PEGylation impairs *in vitro* activity, *in vivo* experiments show that different sizes of PEG can affect liver transduction in different ways and allow liver de-targeting. In fact, low molecular weight PEG does not significantly change the biodistribution profile of PEGylated adenoviral vectors, which continue to accumulate in the liver and to transduce hepatocytes [50]. However, high molecular weight PEG can reduce liver transduction [49]. Another study showed that coating the vector with HPMA polymer results in a 100-fold increase in hepatic transgene expression, resulting in lower liver toxicity and increased vector bioavailability [52]. Covalent attachment of chitosan to adenoviral capsids via a thioether linkage between chitosan modified with 2-iminothiolane and Ad cross-linked with *N*-[c-maleimidobutyryloxy]succinimide ester (GMBS) represents an alternative method for vector coating, allowing transduction into corneal epithelial cells *in vitro* [53].

De-targeting can also be achieved using cationic polyamidoamine (PAMAM) cascade dendrimers that feature high levels of transfection into a wide variety of cellular types in cell culture [54]. Dendrimers represent a novel class of polymers characterized by regular dendritic branching and radial symmetry. A recent study showed that coating Ad with cation PAMAM dendrimers can successfully re-target viral vectors. *In vitro* assays showed increased transduction of cells with low CAR levels and also a slight increase of transduction into hepatic cells (high CAR levels). Nevertheless, excessive coating led to a decrease in transduction levels into cells [55]. In addition, the PAMAM coating also showed increased resistance to NAbs.

An interesting approach is represented by the attachment of superparamagnetic nanoparticles (MNPs) to the surfaces of viral vectors. Cell cultures can be incubated with MNPs/vector complexes formed by electrostatic and hydrophobic interactions and positioned above an external magnetic field gradient generated by specific permanent magnets. MPNs have been used in combination with different viral vectors, such as adenoviral vectors [56], adeno-associated viral vectors [57], and lentiviral vectors [57]. Magnetic beads can be a useful tool for *in vitro* or *ex vivo* cell engineering because the binding of MNPs possibly results in a lower vector dose for optimal transgene delivery while improving transduction efficiency [58].

### 3.3. Active Re-Targeting with Peptide Motifs

Using peptides to actively re-target viral vectors has proven to be a noteworthy approach. New high-throughput screening methods discovered tumor-targeting peptides that have the ability to accumulate in tumors, and for this reason, these peptides may be used to drive viral vectors towards the tumor site. These tumor-homing peptides have been used in different approaches.

For example, peptide-phage library screening revealed novel ligands that are associated with tumor neovascularization, such as the CGKRK peptide. This ligand accumulates on the surface of tumor vessels after intravenous injection, suggesting this peptide is a potential active targeting ligand [59]. Hence, the CGKRK peptide has been tested for its ability to re-target PEGylated adenoviral vectors: PEG molecules, with a maleimide functional group, are attached to the surface of adenoviral vectors; then, a reaction between the SH group of the CGKRK peptide and the functional group of PEG causes covalent binding of the tumor homing peptide to the PEGylated vector [60]. *In vivo* experiments show that after i.v. administration of Ad-PEG-CGKRK into B16BL6-bearing mice, the amount of Ad-PEG-CGKRK in tumor tissue was, respectively, 60-fold and 3-fold higher than that of unmodified Ad and Ad-PEG. In addition, the amount of Ad-PEG-CGKRK in liver tissue was 15-fold less than that of unmodified Ad, but almost the same as Ad-PEG [61]. Interestingly, tumor-homing peptides can be employed not only in therapeutic approaches but also in viral vector-based imaging systems. An example of this is the construction of an adenoviral vector carrying the herpes simplex virus 1 thymidine kinase (HSV1-tk), an enzyme able to metabolize substrates labeled with positron emitter radioisotopes, such as 18F and 124I, that can be visualized by a Positron emission tomography (PET) imaging system. This viral vector has been coated with RGD peptides using PEG linkers. RGD peptides are well known for binding integrins, such as αvβ3, which are usually overexpressed on tumor cells and the neovasculature of tumors of various origins. This engineered adenoviral vector has shown decreased liver uptake and increased tumor accumulation when visualized by a PET imaging system *in vivo* [62].

In addition to enhancing tumor tropism, RGD peptides have also been used for increasing transduction efficiency *in vitro*. In fact, another study focused on the ability of cyclic-RGD peptides to enhance adenoviral transduction into human mesenchymal stem cells (hMSCs). More specifically, hMSCs were incubated with a *GFP*-expressing adenovirus and cyclic-RGD peptides. Fluorescence analysis of this system showed a 4.9-fold increase in gene expression in comparison to hMSCs incubated with the adenovirus alone [63].

### 3.4. Active Re-Targeting with Natural Ligands

In addition to small peptides motifs, real ligands, such as EFG and FGF, the receptors of which are commonly upregulated on the surface of different tumors, have also been employed for tumor targeting [64]. Ligands can be attached to different reactive polymers and the resulting complexes can be used for viral vector coating. Berg *et al.* reported that EGFR-targeted Ad is able to transduce EGFR-positive cells in a specific and CAR-independent way. The polymer poly(2-(dimethylamino)ethyl methacrylate) (pDMAEMA)-avidin has been mixed with biotin-EGF and used for Ad coating in combination with pDMAEMA-PEG complexes for the better charge shielding of Ad. As reported, Ad-pDMAEMA-EGF-PEG successfully transduces EGFR positive cells and CAR-deficient cells. Interestingly, these coated viruses also showed that the light treatment of target cells could enhance transduction efficiency due to photochemical internalization of Ad complexes [65]. Seymour *et al.* used a similar approach. Murine EGF was conjugated to reactive HPMA copolymer and the mEGF-HPMA was used to coat the Ad; this polymer-coated virus showed improved and selective transduction into EGFR positive cells. The *in vivo* performance of an oncolytic adenovirus coated with mEGF-HPMA was illustrated in a murine xenograft model. Mice that were administered mEGF-HPMA-Ad had significantly longer median survival compared with mice that received untargeted HPMA-Ad [66].

Dendrimers can also function as molecular adaptors to bind targeting peptides on the surface of vectors. An EGF mimetic peptide (GE11) has been used to enhance transduction of EGFR overexpressing tumors, which is known to be overexpressed on approximately 60% of solid tumors. The peptide has been linked to the PAMAM dendrimer polymer through a PEG linker and then the adenoviral vector has been coated with the PAMAM-PEG-GE11 complex. Transgene expression analysis revealed a 2.3-fold increase compared to a control vector (Ad-PAMAM-PEG-Cys) *in vitro* [55]. A PAMAM coating has also been employed to engineer the surface of baculovirus-based vectors, which have been used to transduce the *VEGF* gene to stem cells [67]. *In vivo* imaging tests confirmed the role of PAMAM in vector re-targeting using an adenovirus encoding NIS, a theranostic agent used for the noninvasive imaging of biodistribution and transduction efficiency by 123I scintigraphy. Imaging data revealed that viral evasion of liver accumulation led to reduced liver toxicity [68].

EGFR targeting is not the only example of receptor targeting of vectors. Folate receptor is expressed by different types of carcinomas, such as ovarian cancer [69] and nasopharyngeal and laryngeal carcinoma [70]. A simple reaction between naked viral vectors and reactive folate is enough to produce a folate-coated viral vector. This approach has been tested for moloney leukemia virus and adenovirus producing positive results with regards to re-targeting. In fact, after folate conjugation, both vectors were able to transduce human KB cells [71].

Another recent study investigated the effect of a chitosan-PEG-folate complex coating upon the biodistribution of an oncolytic adenovirus. As expected, the additional presence of PEG increased the blood circulation time of oncolytic Ad-nanocomplexes, showing a remarkable 378-fold decrease in liver uptake compared to naked Ad. In addition, this coating strategy resulted in a 2007-fold increase in the tumor-to-liver ratio of oncolytic Ad, leading to a better anti-tumor response and lower hepatic toxicity after i.v. administration [72].

Ligand-based re-targeting has also been possible using the fibroblastic growth factor 2 (FGF2) molecule to target FGFR positive malignant cells. The adenovirus vector has been directly conjugated to FGF2 and the system proved to be effective *in vivo* using either CAR positive or CAR negative cell lines in murine xenograft tumor models [73]. As reported for other ligand molecules, FGF2 can also be attached to reactive polymers, such as PEG [74] or pHPMA [75], resulting in increased blood circulation.

Interestingly, FGF2 retargeted Ad showed increased and unexpected binding to murine blood cells [75]. This evidence suggests that ligands should always be selected carefully and non-specific interactions may occur *in vivo*, leading to reduced availability of injected vectors.

## 4. Conclusions

Many different and interesting strategies have been described in this review. In sum, these new methodologies are a clear sign that gene therapy with viral vectors has been evolving rapidly over the past few years and has taken advantage of new technologies. We can deduce that the viral vector engineering field increasingly depends on research in other related and unrelated fields, especially immunology, biomaterials, and nanoparticles research.

Since the first use of PEG, many other polymers have been individuated as alternative biomaterials to coat vectors, leading to a great variety of possibilities to engineer the surfaces of viral vectors and to offer flexibility to research groups. Polymers conjugated to viral vectors have proven to represent an excellent strategy to hide the vector from the immune system, resulting in lower immunogenicity and reduced risks for human experimentation. In addition, the polymer coating can cause liver de-targeting, avoiding the depletion and fast clearance of vectors from blood. Furthermore, these polymers have also been used as adaptors to bind small molecules to vectors. Peptides and ligands have made possible the active re-targeting of viral vectors to other tissues or tumors.

With further progress in biomaterials and nanoparticle science, a great number of new approaches are expected to emerge that improve old gene delivery systems and create new ones.

## References

[1] Kay A.M., Glorioso G., Naldini L. (2001). Viral vectors for gene therapy: The art of turning infectious agents into vehicles of therapeutics. Nat. Med..

[2] Young L.S., Searle P.F., Onion D., Mautner V. (2006). Viral gene therapy strategies: From basic science to clinical application. J. Pathol..

[3] Escors D., Breckpot K. (2010). Lentiviral vectors in gene therapy: Their current status and future potential. Arch. Immunol. Ther. Exp..

[4] Palmer D.J., Ng P. (2011). Characterization of helper-dependent adenoviral vectors. Cold Spring Harb. Protoc..

[5] Brunetti-Pierri N., Ng P. (2011). Helper-dependent adenoviral vectors for liver-directed gene therapy. Hum. Mol. Genet..

[6] Cooray S., Howe S.J., Thrasher A.J. (2012). Retrovirus and lentivirus vector design and methods of cell conditioning. Methods Enzymol..

[7] Miyoshi H., Blomer U., Takahashi M., Gage F.H., Verma I.M. (1998). Development of a self-inactivating lentivirus vector. J. Virol..

[8] Brunetti-Pierri N., Palmer D.J., Beaudet A.L., Carey K.D., Finegold M., Ng P. (2004). Acute toxicity after high-dose systemic injection of helper-dependent adenoviral vectors into nonhuman primates. Hum. Gene Ther..

[9] Muruve D.A., Cotter M.J., Zaiss A.K., White L.R., Liu Q., Chan T., Clark S.A., Ross P.J., Meulenbroek R.A., Maelandsmo G.M. (2004). Helper-dependent adenovirus vectors elicit intact innate but attenuated adaptive host immune responses *in vivo*. J. Virol..

[10] Yamaguchi T., Kawabata K., Koizumi N., Sakurai F., Nakashima K., Sakurai H., Sasaki T., Okada N., Yamanishi K., Mizuguchi H. (2007). Role of Myd88 and TLR9 in the innate immune response elicited by serotype 5 adenoviral vectors. Hum. Gene Ther..

[11] Martinez J., Huang X., Yang Y. (2010). Toll-like receptor 8-mediated activation of murine plasmacytoid dendritic cells by vaccinia viral DNA. Proc. Natl. Acad. Sci. USA.

[12] Cerullo V., Seiler M.P., Mane V., Brunetti-Pierri N., Clarke C., Bertin T.K., Rodgers J.R., Lee B. (2007). Toll-like receptor 9 triggers an innate immune response to helper-dependent adenoviral vectors. Mol. Ther..

[13] Seiler M.P., Cerullo V., Lee B. (2007). Immune response to helper dependent adenoviral mediated liver gene therapy: Challenges and prospects. Curr. Gene Ther..

[14] Breckpot K., Escors D., Arce F., Lopes L., Karwacz K., van Lint S., Keyaerts M., Collins M. (2010). HIV-1 lentiviral vector immunogenicity is mediated by toll-like receptor 3 (TLR3) and TLR7. J. Virol..

[15] Suzuki M., Cerullo V., Bertin T.K., Cela R., Clarke C., Guenther M., Brunetti-Pierri N., Lee B. (2010). Myd88-dependent silencing of transgene expression during the innate and adaptive immune response to helper-dependent adenovirus. Hum. Gene Ther..

[16] Rhee E.G., Blattman J.N., Kasturi S.P., Kelley R.P., Kaufman D.R., Lynch D.M., La Porte A., Simmons N.L., Clark S.L., Pulendran B. (2011). Multiple innate immune pathways contribute to the immunogenicity of recombinant adenovirus vaccine vectors. J. Virol..

[17] Pradere J.P., Dapito D.H., Schwabe R.F. (2013). The yin and yang of toll-like receptors in cancer. Oncogene.

[18] Alba R., Bradshaw A.C., Coughlan L., Denby L., McDonald R.A., Waddington S.N., Buckley S.M., Greig J.A., Parker A.L., Miller A.M. (2010). Biodistribution and retargeting of FX-binding ablated adenovirus serotype 5 vectors. Blood.

[19] Bayo-Puxan N., Gimenez-Alejandre M., Lavilla-Alonso S., Gros A., Cascallo M., Hemminki A., Alemany R. (2009). Replacement of adenovirus type 5 fiber shaft heparan sulfate proteoglycan-binding domain with RGD for improved tumor infectivity and targeting. Hum. Gene Ther..

[20] Edelstein M.L., Abedi M.R., Wixon J., Edelstein R.M. (2004). Gene therapy clinical trials worldwide 1989–2004—An overview. J. Gene Med..

[21] Raper S.E., Chirmule N., Lee F.S., Wivel N.A., Bagg A., Gao G.-P., Wilson J.M., Batshaw M.L. (2003). Fatal systemic inflammatory response syndrome in a ornithine transcarbamylase deficient patient following adenoviral gene transfer. Mol. Genet. Metab..

[22] Schulte M., Sorkin M., Al-Benna S., Stupka J., Hirsch T., Daigeler A., Kesting M.R., Steinau H.U., Jacobsen F., Steinstraesser L. (2013). Innate immune response after adenoviral gene delivery into skin is mediated by AIM2, NALP3, DAI and MDA5. SpringerPlus.

[23] Veronese F.M., Mero A. (2008). The impact of pegylation on biological therapies. Biodrugs.

[24] Croyle M.A., Le H.T., Linse K.D., Cerullo V., Toietta G., Beaudet A., Pastore L. (2005). Pegylated helper-dependent adenoviral vectors: Highly efficient vectors with an enhanced safety profile. Gene Ther..

[25] Wonganan P., Croyle M.A. (2010). Pegylated adenoviruses: From mice to monkeys. Viruses.

[26] Tesfay M.Z., Kirk A.C., Hadac E.M., Griesmann G.E., Federspiel M.J., Barber G.N., Henry S.M., Peng K.W., Russell S.J. (2013). Pegylation of vesicular stomatitis virus extends virus persistence in blood circulation of passively immunized mice. J. Virol..

[27] Alemany R., Suzuki K., Curiel D.T. (2000). Blood clearance rates of adenovirus type 5 in mice. J. Gen. Virol..

[28] Metzner C., Kochan F., Dangerfield J.A. (2013). Postexit surface engineering of retroviral/lentiviral vectors. BioMed Res. Int..

[29] Leggiero E., Astone D., Cerullo V., Lombardo B., Mazzaccara C., Labruna G., Sacchetti L., Salvatore F., Croyle M., Pastore L. (2013). Pegylated helper-dependent adenoviral vector expressing human Apo A-I for gene therapy in ldlr-deficient mice. Gene Ther..

[30] Kreppel F., Kochanek S. (2008). Modification of adenovirus gene transfer vectors with synthetic polymers: A scientific review and technical guide. Mol. Ther..

[31] Eto Y., Yoshioka Y., Ishida T., Yao X., Morishige T., Narimatsu S., Mizuguchi H., Mukai Y., Okada N., Kiwada H. (2010). Optimized pegylated adenovirus vector reduces the anti-vector humoral immune response against adenovirus and induces a therapeutic effect against metastatic lung cancer. Biol. Pharm. Bull..

[32] Lee G.K., Maheshri N., Kaspar B., Schaffer D.V. (2005). PEG conjugation moderately protects adeno-associated viral vectors against antibody neutralization. Biotechnol. Bioeng..

[33] Croyle M.A., Callahan S.M., Auricchio A., Schumer G., Linse K.D., Wilson J.M., Brunner L.J., Kobinger G.P. (2003). PEGylation of a vesicular stomatitis virus G pseudotyped lentivirus vector prevents inactivation in serum. J. Virol..

[34] Ahn C.H., Chae S.Y., Bae Y.H., Kim S.W. (2004). Synthesis of biodegradable multi-block copolymers of poly(l-lysine) and poly(ethylene glycol) as a non-viral gene carrier. J. Control. Release.

[35] Zeng Q., Han J., Zhao D., Gong T., Zhang Z., Sun X. (2012). Protection of adenovirus from neutralizing antibody by cationic PEG derivative ionically linked to adenovirus. Int. J. Nanomed..

[36] Fisher K.D., Seymour L.W. (2010). HPMA copolymers for masking and retargeting of therapeutic viruses. Adv. Drug Deliv. Rev..

[37] Wang C.H., Chan L.W., Johnson R.N., Chu D.S., Shi J., Schellinger J.G., Lieber A., Pun S.H. (2011). The transduction of coxsackie and adenovirus receptor-negative cells and protection against neutralizing antibodies by HPMA-co-oligolysine copolymer-coated adenovirus. Biomaterials.

[38] Kim P.H., Kim J., Kim T.I., Nam H.Y., Yockman J.W., Kim M., Kim S.W., Yun C.O. (2011). Bioreducible polymer-conjugated oncolytic adenovirus for hepatoma-specific therapy via systemic administration. Biomaterials.

[39] Singh R., Al-Jamal K.T., Lacerda L., Kostarelos K. (2008). Nanoengineering artificial lipid envelopes around adenovirus by self-assembly. ACS Nano.

[40] Yilmazer A., Al-Jamal W.T., van den Bossche J., Kostarelos K. (2013). The effect of artificial lipid envelopment of adenovirus 5 (ad5) on liver de-targeting and hepatotoxicity. Biomaterials.

[41] Bergelson J.M., Cunningham J.A., Droguett G., Kurt-Jones E.A., Krithivas A., Hong J.S., Horwitz M.S., Crowell R.L., Finberg R.W. (1997). Isolation of a common receptor for coxsackie b viruses and adenoviruses 2 and 5. Science.

[42] Hastie E., Cataldi M., Marriott I., Grdzelishvili V.Z. (2013). Understanding and altering cell tropism of vesicular stomatitis virus. Virus Res..

[43] Finkelshtein D., Werman A., Novick D., Barak S., Rubinstein M. (2013). LDL receptor and its family members serve as the cellular receptors for vesicular stomatitis virus. Proc. Natl. Acad. Sci. USA.

[44] Glazkova D.V., Vetchinova A.S., Bogoslovskaia E.V., Zhogina Y.A., Markelov M.L., Shipulin G.A. (2013). Downregulation of human *CCR5* receptor gene expression using artificial microRNAs. Mol. Biol..

[45] Martin K., Brie A., Saulnier P., Perricaudet M., Yeh P., Vigne E. (2003). Simultaneous car- and alpha v integrin-binding ablation fails to reduce ad5 liver tropism. Mol. Ther..

[46] Waddington S.N., McVey J.H., Bhella D., Parker A.L., Barker K., Atoda H., Pink R., Buckley S.M., Greig J.A., Denby L. (2008). Adenovirus serotype 5 hexon mediates liver gene transfer. Cell.

[47] Wonganan P., Clemens C.C., Brasky K., Pastore L., Croyle M.A. (2011). Species differences in the pharmacology and toxicology of PEGylated helper-dependent adenovirus. Mol. Pharm..

[48] Matsui H., Sakurai F., Katayama K., Yamaguchi T., Okamoto S., Takahira K., Tachibana M., Nakagawa S., Mizuguchi H. (2012). A hexon-specific PEGylated adenovirus vector utilizing blood coagulation factor x. Biomaterials.

[49] Doronin K., Shashkova E.V., May S.M., Hofherr S.E., Barry M.A. (2009). Chemical modification with high molecular weight polyethylene glycol reduces transduction of hepatocytes and increases efficacy of intravenously delivered oncolytic adenovirus. Hum. Gene Ther..

[50] Mok H., Palmer D.J., Ng P., Barry M.A. (2005). Evaluation of polyethylene glycol modification of first-generation and helper-dependent adenoviral vectors to reduce innate immune responses. Mol. Ther..

[51] Hofherr S.E., Shashkova E.V., Weaver E.A., Khare R., Barry M.A. (2008). Modification of adenoviral vectors with polyethylene glycol modulates *in vivo* tissue tropism and gene expression. Mol. Ther..

[52] Green N.K., Herbert C.W., Hale S.J., Hale A.B., Mautner V., Harkins R., Hermiston T., Ulbrich K., Fisher K.D., Seymour L.W. (2004). Extended plasma circulation time and decreased toxicity of polymer-coated adenovirus. Gene Ther..

[53] Wang I.J., Jhuang M.C., Chen Y.H., Yeh L.K., Liu C.Y., Young T.H. (2010). Chitosan modification of adenovirus to modify transfection efficiency in bovine corneal epithelial cells. PLoS One.

[54] Kang S.H., Zirbes E.L., Kole R. (1999). Delivery of antisense oligonucleotides and plasmid DNA with various carrier agents. Antisense Nucleic Acid Drug Dev..

[55] Vetter A., Virdi K.S., Espenlaub S., Rodl W., Wagner E., Holm P.S., Scheu C., Kreppel F., Spitzweg C., Ogris M. (2013). Adenoviral vectors coated with PAMAM dendrimer conjugates allow car independent virus uptake and targeting to the egf receptor. Mol. Pharm..

[56] Pandori M.W., Hobson D.A., Sano T. (2002). Adenovirus-microbead conjugates possess enhanced infectivity: A new strategy for localized gene delivery. Virology.

[57] Mah C., Fraites T.J., Zolotukhin I., Song S., Flotte T.R., Dobson J., Batich C., Byrne B.J. (2002). Improved method of recombinant AAV2 delivery for systemic targeted gene therapy. Mol. Ther..

[58] Sapet C., Pellegrino C., Laurent N., Sicard F., Zelphati O. (2012). Magnetic nanoparticles enhance adenovirus transduction *in vivo* and *in vivo*. Pharm. Res..

[59] Hoffman J.A., Giraudo E., Singh M., Zhang L., Inoue M., Porkka K., Hanahan D., Ruoslahti E. (2003). Progressive vascular changes in a transgenic mouse model of squamous cell carcinoma. Cancer Cell.

[60] Yao X.L., Yoshioka Y., Ruan G.X., Chen Y.Z., Mizuguchi H., Mukai Y., Okada N., Gao J.Q., Nakagawa S. (2012). Optimization and internalization mechanisms of PEGylated adenovirus vector with targeting peptide for cancer gene therapy. Biomacromolecules.

[61] Yao X., Yoshioka Y., Morishige T., Eto Y., Narimatsu S., Kawai Y., Mizuguchi H., Gao J.Q., Mukai Y., Okada N. (2011). Tumor vascular targeted delivery of polymer-conjugated adenovirus vector for cancer gene therapy. Mol. Ther..

[62] Xiong Z., Cheng Z., Zhang X., Patel M., Wu J.C., Gambhir S.S., Chen X. (2006). Imaging chemically modified adenovirus for targeting tumors expressing integrin alphavbeta3 in living mice with mutant herpes simplex virus *type 1 thymidine kinase* pet reporter gene. J. Nucl. Med..

[63] King W.J., Krebsbach P.H. (2013). Cyclic-RGD peptides increase the adenoviral transduction of human mesenchymal stem cells. Stem Cells Dev..

[64] Black P.C., Agarwal P.K., Dinney C.P. (2007). Targeted therapies in bladder cancer–An update. Urol. Oncol..

[65] Bonsted A., Engesaeter B.O., Hogset A., Maelandsmo G.M., Prasmickaite L., D’Oliveira C., Hennink W.E., van Steenis J.H., Berg K. (2006). Photochemically enhanced transduction of polymer-complexed adenovirus targeted to the epidermal growth factor receptor. J. Gene Med..

[66] Morrison J., Briggs S.S., Green N., Fisher K., Subr V., Ulbrich K., Kehoe S., Seymour L.W. (2008). Virotherapy of ovarian cancer with polymer-cloaked adenovirus retargeted to the epidermal growth factor receptor. Mol. Ther..

[67] Paul A., Shao W., Abbasi S., Shum-Tim D., Prakash S. (2012). Pamam dendrimer-baculovirus nanocomplex for microencapsulated adipose stem cell-gene therapy: *In vitro* and *in vivo* functional assessment. Mol. Pharm..

[68] Grunwald G.K., Vetter A., Klutz K., Willhauck M.J., Schwenk N., Senekowitsch-Schmidtke R., Schwaiger M., Zach C., Wagner E., Goke B. (2013). Systemic image-guided liver cancer radiovirotherapy using dendrimer-coated adenovirus encoding the sodium iodide symporter as theranostic gene. J. Nucl. Med..

[69] Walters C.L., Arend R.C., Armstrong D.K., Naumann R.W., Alvarez R.D. (2013). Folate and folate receptor alpha antagonists mechanism of action in ovarian cancer. Gynecol. Oncol..

[70] Xie M., Zhang H., Xu Y., Liu T., Chen S., Wang J., Zhang T. (2013). Expression of folate receptors in nasopharyngeal and laryngeal carcinoma and folate receptor-mediated endocytosis by molecular targeted nanomedicine. Int. J. Nanomed..

[71] Reddy J.A., Clapp D.W., Low P.S. (2001). Retargeting of viral vectors to the folate receptor endocytic pathway. J. Control. Release.

[72] Kwon O.J., Kang E., Choi J.W., Kim S.W., Yun C.O. (2013). Therapeutic targeting of chitosan-peg-folate-complexed oncolytic adenovirus for active and systemic cancer gene therapy. J. Control. Release.

[73] Wang W.J., Zhu N.L., Chua J., Swenson S., Costa F.K., Schmitmeier S., Sosnowski B.A., Shichinohe T., Kasahara N., Chen T.C. (2005). Retargeting of adenoviral vector using basic fibroblast growth factor ligand for malignant glioma gene therapy. J. Neurosurg..

[74] Lanciotti J., Antonius S., Doukas J., Sosnowski B., Pierce G., Gregory R., Wadsworth S., O’Riordan C. (2003). Targeting adenoviral vectors using heterofunctional polyethylene glycol fgf2 conjugates. Mol. Ther..

[75] Green N.K., Morrison J., Hale S., Briggs S.S., Stevenson M., Subr V., Ulbrich K., Chandler L., Mautner V., Seymour L.W. (2008). Retargeting polymer-coated adenovirus to the FGF receptor allows productive infection and mediates efficacy in a peritoneal model of human ovarian cancer. J. Gene Med..

